# Characterizing Pure Polymers under High Speed Compression for the Micromechanical Prediction of Unidirectional Composites

**DOI:** 10.3390/polym15051262

**Published:** 2023-03-02

**Authors:** Pei Hao, Siebe W. F. Spronk, Ruben D. B. Sevenois, Wim Van Paepegem, Francisco A. Gilabert

**Affiliations:** 1 Department of Materials, Textiles and Chemical Engineering (MaTCh), Mechanics of Materials and Structures (MMS), Tech Lane Ghent Science Park—Campus A, Ghent University (UGent), Technologiepark-Zwijnaarde 46, Zwijnaarde, 9052 Ghent, Belgium; 2 SIM Program M3, Technologiepark Zwijnaarde 48, Zwijnaarde, 9052 Ghent, Belgium; 3 Solvay Materials Science Application Center, Ransbeekstraat 310, 1120 Brussels, Belgium

**Keywords:** high strain rate, dynamic compression, PEEK, epoxy, micro-thermomechanics, RVE modelling, finite element analysis

## Abstract

The nonlinear behaviour of fibre-reinforced polymer composites (FRPC) in transverse loading is mainly induced by the constituent polymer matrix. The thermoset and thermoplastic matrices are typically rate- and temperature-dependent, complicating the dynamic material characterization process. Under dynamic compression, the microstructure of the FRPC develops local strains and local strain rates whose values can be much higher than those applied at macroscopic level. The correlation between the local (microscopic) values and the measurable (macroscopic) ones still present challenges when applying the strain rate in the range 10${}^{-3}$–10${}^{3}$ s${}^{-1}$. This paper presents an in-house uniaxial compression test setup to provide robust stress–strain measurements applying strain rates up to 100 s${}^{-1}$. A semi-crystalline thermoplastic polyetheretherketone (PEEK) and a toughened thermoset epoxy PR520 are assessed and characterized. The thermomechanical response of the polymers is further modelled using an advanced glassy polymer model, naturally capturing the isothermal to adiabatic transition. A micromechanical model of a unidirectional composite undergoing dynamic compression is developed by using both validated polymers as matrices reinforced by carbon fibres (CF) using Representative Volume Element (RVE) models. These RVEs are used to analyse the correlation between the micro- and macroscopic thermomechanical response of the CF/PR520 and CF/PEEK systems investigated at intermediate to high strain rates. Both systems experience an excessive strain localization with local plastic strain about 19% when a macroscopic strain of 3.5% is applied. The comparison of using a thermoplastic and a thermoset as a matrix in composites is discussed with regard to the rate-dependence, the interface debonding and the self-heating effect.

## 1. Introduction

The thermomechanical performance of fibre-reinforced polymer composites (FRPC) relies heavily on the interaction and properties of the constituents: fibre, polymer matrix and interface. The nonlinear thermomechanical behaviour of the polymer matrix greatly affects the overall rate- and temperature-response of the composite, particularly under transverse loading. The stress–strain response of the polymer matrix can differ significantly between thermosets and thermoplastics. The composite system based on an epoxy matrix has been widely studied experimentally and numerically [1,2,3,4,5]. Recently, thermoplastic-based composites keep replacing the traditional epoxy counterparts thanks to their recyclability. Thermoplastic composites also show excellent damage and impact resistance [6], but they are more sensitive to temperature and moisture [7]. The investigation of different thermoplastic composites has been carried out from high performance polyetheretherketone (PEEK) [1] to commodity polymers, for instance, polypropylene (PP) [8,9] and polyamide6 (PA6) [10,11].

Both thermoset and thermoplastic composite systems show a significant rate sensitivity under different loading conditions: transverse tension and compression [2,12], longitudinal compression [13], off-axis compression [14] and transverse tension/in-plane shear loadings using off-axis tension specimens [4]. Unidirectional (UD) composites have been investigated extensively by researchers. The quasi-static response of UD composites and polymers in their pure state can be obtained by universal testing machines, whereas the dynamic test generally relies on the Kolsky bar method [8,15]. Under quasi-static loading conditions, Sommer et al. [9] conducted in situ experiments to study the damage mechanisms in glass fibre (GF)-reinforced polypropylene (PP), and different damage modes were reported: matrix cracking, delamination and fibre breakage. Depending on the applied strain rate, Koyanagi et al. [16] experimentally studied the failure modes. It was found that an interface-failure-dominant mode occurs at a relatively high strain rate, and a matrix-failure-dominant mode at a relatively low strain rate. Kim et al. [8] investigated the effects of the strain rate on a GF/PP composite based on a split-Hopkinson pressure bar and reported that the failure strengths were significantly increased with the strain rates. Hsiao and Daniel [1] compared two carbon fibre (CF) reinforced UD composite systems (i.e., CF/epoxy and CF/PEEK laminates). Both systems show a similar trend of the rate-dependent strength, which increases as the applied strain rate increases. It was believed that the transverse strength in the UD composite results from the viscoelasticity of polymers and the time-dependent nature of progressive damage. However, the excessive strain localization occurring at a relative small global strain was often overlooked due to the lack of microscale observation. Because of the random fibre distribution, strain localization can be generally observed outside the resin pocket where the matrix ligaments are formed due to the fibre–matrix interface debonding. These polymer ligaments are prone to undergo large plastic deformations. The literature on rate-sensitivity for pure polymer and composites is mostly concerned with treating low rates, extremely high rates, or treating both. Only a few researchers carried out the studies from the intermediate to high strain rate range due to testing difficulties. Shokrieh and Omidi [12] characterized the transverse tensile and compressive properties of unidirectional GF/epoxy at intermediate strain rates of 1–100 s${}^{-1}$ using a high-speed servo-hydraulic machine. Spronk [17] investigated extensively two composite systems relevant for the automotive industry: CF/epoxy and GF/PA6. A hydraulic pulse test bench was employed for the dynamic tensile tests with a strain rate up to 200 s${}^{-1}$.

Transverse loading is one of the most critical loading cases for UD composites because the nonlinearity of the polymer matrix plays an important role in the thermomechanical response. Theoretical and numerical methods have been widely used for the stress analysis of composites [18,19], and the recently proposed ‘Differential Quadrature’ [20] and ‘Bezier’ [21] methods proved to have high stability and accuracy. Alternatively, computational micromechanical studies are frequently based on finite element (FE) simulations using representative volume elements (RVE). The microstructure of the composite and the attributes of each constituent are prerequisite. Koyanagi et al. [2] generated a periodic unit-cell containing 20 fibres to study the strain-rate dependent transverse tensile failure of CF/epoxy. An elasto-viscoplastic constitutive equation with continuum damage mechanics was used for the epoxy matrix. Garoz et al. [22] performed a systematic study using new proposed periodic boundary conditions (PBC) for conformal and non-conformal meshes in the micromechanical model of a UD CF/epoxy composite. To understand the mechanism in microscale, Chevalier et al. [5] have conducted μ-DIC using a micro-notched specimen of CF/epoxy via a scanning electron microscope (SEM), showing ththe maximum local strain attained was 20% under transverse compression. The RVE simulation was carried out based on a constitutive model validated for a bulky epoxy at different strain rates. Sharma et al. [23] used a linear Drucker--Prager plasticity model to characterize the epoxy matrix and conducted RVE simulations combined with PBCs. Ullah et al. [3] used an elasto-plastic material based on a non-associative pressure dependent paraboloidal yield criterion to investigate different unidirectional FRP and textile composites. Melro et al. [24] has proposed a plastic-damage material continuum model for a typical epoxy matrix and further used it for the micromechanical analyses of composite materials. This model and its extension have been widely used by other researchers for micromechanical studies: the effect of ply thickness on the transverse compressive strength [25] and estimation of the mode I through-thickness intralaminar crack resistance curve [26].

However, it is noted that most micromechanical studies rely on a rate-independent model for epoxy matrix. The macroscopical observed rate-dependence of UD composites cannot be accurately characterized. Therefore, this paper proposes a complete work-flow for the thermomechanical modelling of UD composites considering the origin of rate-dependence from polymer matrices. It is also well-known that polymers are rate- and temperature-dependent, as well as presenting a prominent self-heating and thermal softening effects at an elevated strain rate [27,28]. A fully coupled thermomechanical FEM-based framework was implemented accounting for the semi-crystalline polymers (SCP) [29]. The proposed Unified SCP (USCP) model was developed in a modular way to extend its applicability to a broad variety of thermosets or thermoplastics validated at different strain rates and temperatures. Recently, based on the USCP model, Hao et al. [30] developed an experimental-modelling combined approach to compare a toughened epoxy PR520 and a PEEK across the intermediate to high strain rate range.

In this paper, the USCP model was applied to investigate the thermomechanical response of UD composites under high-speed transverse compression. Two composite systems are selected: a CF/PR520 and a high performance CF/PEEK. Section 2 presents the in-house compressive bench to test polymers under high speed. This section also presents the selected commercial polymers (a toughened epoxy CYCOM^®^ PR520 and KetaSpire^®^ KT880−NT), the testing conditions and the polymer response. Section 3 describes the constitutive equations of the polymer model used to capture both types of polymers as well as its validation at different strain rates. Section 4 introduces the RVE-based micromechanical model representing a UD CFRP that shows the applicability of the polymer model and the results from the test bench. Section 5 is devoted to put in practice the RVE model using the characterized polymers under compression. This section presents the model prediction of the two UD composite systems under a very high compression rate, where rate-dependence, fibre debonding and matrix self-heating are investigated at global and local scales.

## 2. Experimental Methodology

This section describes an in-house hydraulic pulse test bench to conduct high speed compression tests using cylinder-shaped specimens, in which two types of polymers are used. The required recording instrumentation to capture both thermal and mechanical responses is also discussed. The resulting stress–strain compression curves from both polymer systems are described and discussed. Using infrared camera inspection, this section also shows temperature evolution in the specimens and its correlation to the stress–strain curve.

### 2.1. High Speed Compression Setup

**Figure 1:**
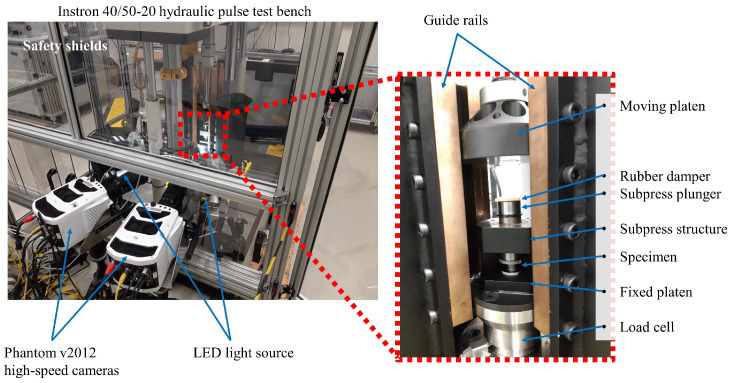
High speed compression setup based on the Instron 40/50-20 hydraulic pulse test bench accompanied with the high-speed cameras, lighting and guide rails.

To conduct compressive tests at rates up to 100 s${}^{-1}$, a hydraulic pulse test bench of an Instron 40/50-20 has been used. This device is capable of reaching a maximum test speed of 20 m/s with a load capacity of 50 kN. Figure 1 shows the in-house guide rail structure with linear bearings, which prevents the possible sideways movement of the compression platen connected to the machine actuator. The upper moving compression platen moves freely for a predetermined distance to attain a constant velocity. This prevents the effect of the speed-up of the actuator that might falsify the prescribed strain rate at which the specimen is deformed. To provide the best trade-off between attenuating impact vibration and quickly accelerating the subpress piston, a piece of 2 mm-thick stiff rubber is placed on top of the subpress.

In terms of instrumentation, two synchronized Phantom v2012 high-speed cameras in a main–secondary setup operate at 20,000 fps with a 1280 × 800 monochrome resolution at a bit depth of 12 bits. High-intensity DIC LED lighting and polarizers on the lights were used. To achieve a throughput of 200 fps, the FLIR A655sc infrared camera run at a reduced resolution of 640 × 120 pixels. The force and displacement signals were exported from the test bench through the NI USB-6363 DAQ card. The acquisition was achieved with a maximum speed of 2 M Samples cumulated across all analogue channels. Automatic synchronization of the images was carried out in the Phantom Camera Control v3.6 software, which is based on a parallel digital acquisition of the signals produced by the master camera. The Kistler 9071A piezoelectric loadcell, read out using a Kistler 5015A charge amplifier, was calibrated together to give a 10 V output at 50 kN (using a 50 kN Instron quasi-static loadcell for an in-series calibration). In the current case, the large amount of deformation leads to relatively long test durations with a smooth force signal. A long time constant is therefore chosen for the charge amplifier, limiting the signal decay to a minimum, while the inevitable related reduction of the acquisition bandwidth does not pose a problem.

### 2.2. Materials and Testing Conditions

**Figure 2:**
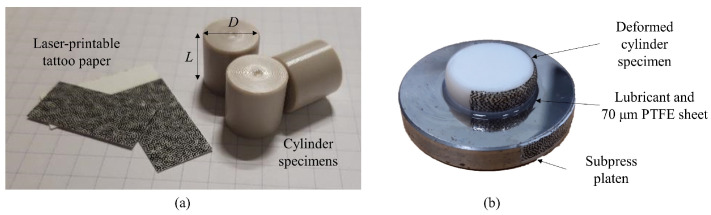
Specimen preparation: (**a**) machined cylinder compression specimens and laser-printed speckle pattern film; and (**b**) consistent deformation of a specimen and DIC pattern under large strain.

A semi-crystalline thermoplastic PEEK and a toughened thermoset epoxy are chosen as representative polymers. The PEEK corresponds to the commercial KetaSpire^®^ KT880−NT supplied in pellet form and shaped to rods through an injection moulding process. Specimens were obtained by machining to the same diameter *D* and length *L* (i.e., 12 mm) with high accuracy as Figure 2a shows. The specimens of epoxy were made of CYCOM^®^ PR520 resin and they were fully cured following a 2-h dwell at 180 °C, to be later machined as cylinders with the same diameter and length as 10 mm. All specimens in this work were pre-conditioned in a relative humidity of 50% for at least 24 h.

Figure 2b presents the application of a DIC speckle pattern using a laser-printable tattoo paper, which allows following large deformations on non-flat surfaces. A second set of DIC tattoo papers are also attached to the surface of the rigid compression platens to track the movement of the cross-head for calculating the true strain rate. The diameter of the subpress platen is small enough such that its DIC pattern remains relatively sharp despite being slightly out of focus.

In terms of loading conditions, a series of high-speed monotonic uniaxial compression tests were performed at loading speeds of the cross-head of 0.01 m/s and 1 m/s at room temperature (RT). These loading rates typically represent the thermal-coupled and nearly adiabatic scenarios, respectively.

**Figure 3:**
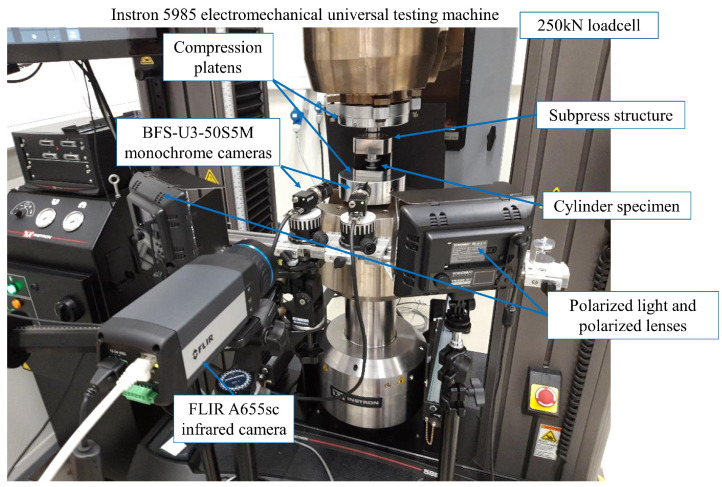
Subpress fixture mounted in Instron 5985 electromechanical test bench that equips DIC and temperature measuring instrument.

Additionally, quasi-static (QS) compression tests were also carried out at RT using an Instron 5985 electromechanical universal testing machine. Figure 3 presents the experimental setup that equips two monochrome cameras, an infrared camera and a data acquisition system. The loading speeds of the cross-head were chosen as 5 mm/min for PEEK KT880−NT and 2 mm/min for PR520. The low loading rates ensure the QS test is in an isothermal loading condition, where surface temperature measurements were recorded by the 640 × 480 pixel infrared camera. The monochrome cameras with 5 megapixels were focused at the cylinder compression specimen for the stereo-DIC. The specimen was loaded through two compression platens, which were self-aligned at 100 kN. A 250 kN loadcell was connected. A NI USB-6363 DAQ card was also connected to acquire the force and displacement signal exported from the test bench.

### 2.3. Data Acquisition

**Figure 4:**
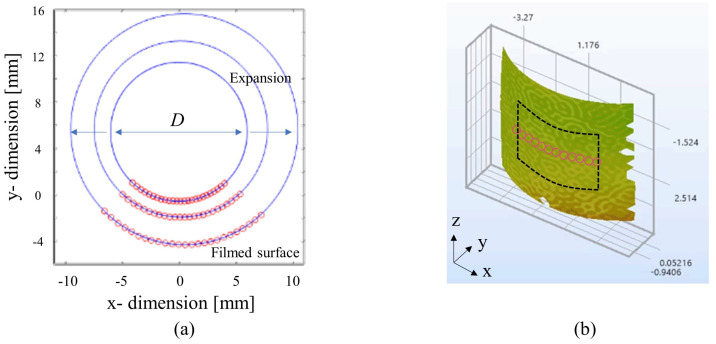
Filmed deformed specimen surface represented by (**a**) the evolution of the cross−sectional surface area with the fit of circle onto horizontal mid−plane and (**b**) vertical Hencky strain post-processed by 3D stereo−DIC system.

The force was directly sampled from the test bench. The displacement was tracked using the optical extensometer between the two platens. The strain field was measured using a 3D DIC analysis of Figure 4. The horizontal expansion of the mid-plane of the cylinder was tracked as shown in Figure 4a. Figure 4b shows the procedure to obtain the average vertical Hencky strain, considering 5 × 5 mm${}^{2}$ in the middle of the filmed specimen surface.

### 2.4. Polymer Response

**Figure 5:**
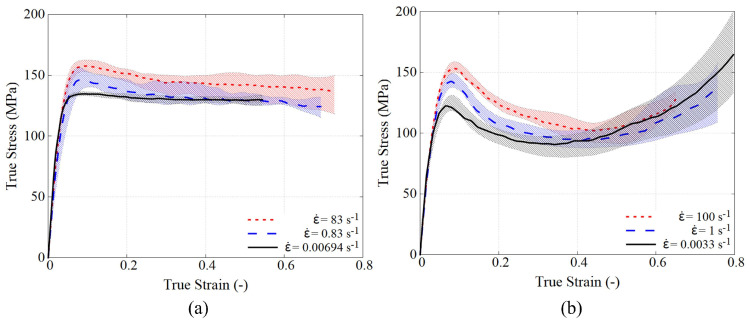
Experimental results under compression at different strain rates at 25 °C: (**a**) PEEK KT880−NT and (**b**) PR520.

Figure 5 shows the averaged true stress–strain curves for PEEK and PR520. In order to obtain a representative trend, each test was repeatedly conducted for at least five times. The shadow in each curve gives the upper and lower bounds from the repetitions at that load condition.

The elastic modulus is obtained from the initial straight slope, giving a value of 4.334 GPa for PEEK and 4.278 GPa for PR520. According to the analysed data, the viscoelastic response in both polymers is barely observed. Both polymers exhibit a clear peak rate-dependent yield stress, and similar peak yield stresses are found for both polymers at different strain rates. After the peak stress, PEEK develops a relatively constant plastic flow. However, PR520 presents a prominent post-peak strain softening followed by a remarkable nonlinear stress increase beyond the strain of 0.4. This nonlinear increase, also observed by other authors [31], is due to the rubbery effect causing reorientation of the polymer chains, which dominates the material response at the large strain range [32]. PEEK does not show this rubbery response because its molecular chains are less capable of generating this reorientation in the investigated strain range.

The stress–strain curves in both polymers at high strain rate experience a dropping trend that intersects their response at a lower strain rate. This behaviour is attributed to the thermal softening and has also been reported in another epoxy resin under compression [31]. Under high speed compression, the test duration is very short and heat generated from the plastic dissipation cannot be exchanged with the environment. This non-released heat degrades the material properties causing the softening at large strains.

**Figure 6:**
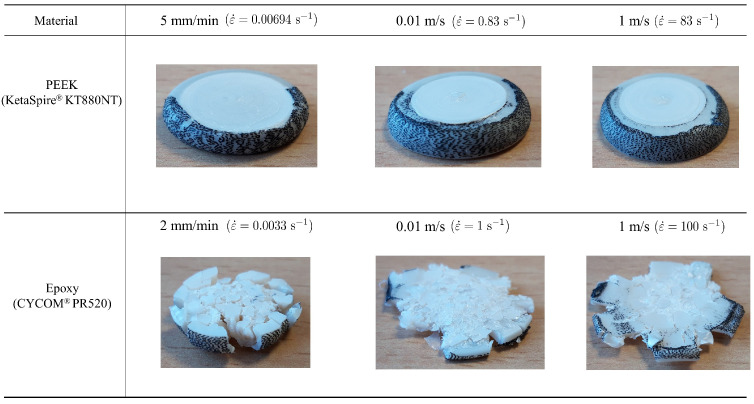
Morphology of samples at different strain rates at the end of the tests: PEEK KT880−NT and PR520.

Figure 6 shows the specimens of PEEK and PR520 after the tests at different strain rates. Although both polymers underwent significant deformation, it is worth mentioning that PEEK did not experience failure at any strain rate and the morphology of both ends is conserved. This can be attributed to the recrystallization process, which caused a change in the microstructure on the ends and edges compared to the inner regions. However, PR520 specimens were crushed, generating a multitude of small fragments in all cases. Figure 6 depicts the crushed PR520 specimens, where the cylinders were fully destroyed and their integrity cannot be preserved as in the PEEK specimens.

### 2.5. Self-Heating Production and Thermal Softening

**Figure 7:**
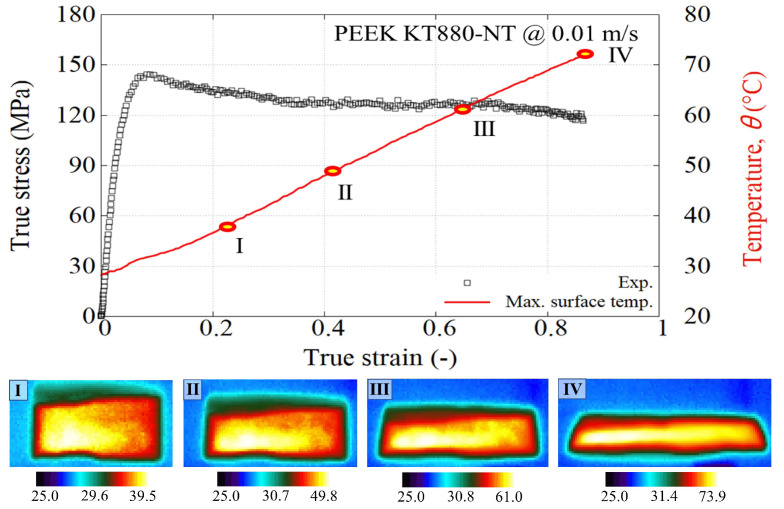
Stress-strain response and temperature evolution of PEEK KT880−NT under compression at 0.01 m/s. Temperature of infrared pictures in °C.

Figure 7 shows four stages captured during the compression of PEEK at 0.01 m/s. It can be seen that the surface peak temperature rises monotonically from RT to 73 °C, which is far below the glass transition temperature, and the polymer is unlikely to soften significantly. The maximum temperature was consistently found in the sample’s mid-plane due to heat conduction to the adjacent subpress platens. It is noticeable how this temperature rises steadily as a function of the true strain. The temperature evolution in the PR520 specimens showed a similar linear trend with the true strain, except for suffering a sudden increase at the failure instant (more details can be consulted in Ref. [30]).

**Figure 8:**
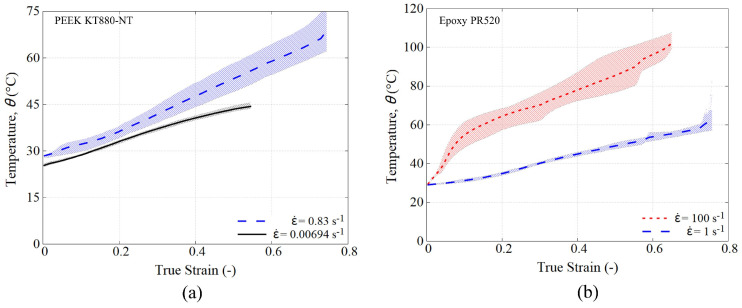
Temperature evolution at different strain rates: (**a**) PEEK KT880−NT and (**b**) PR520.

Figure 8 summarizes, in average form, the temperature evolution with increasing strain. Two sets of strain rates are presented: low-medium for PEEK (0.00694 and 0.83 s${}^{-1}$) and medium-high for PR520 (1 and 100 s${}^{-1}$).

It is interesting to see that, even at the lowest tested strain-rate (0.00694 s${}^{-1}$), the self-heating still takes place. At the fastest tested strain-rate (100 s${}^{-1}$), there is a rapid temperature increase from RT to 58 °C up to the strain of 10 %, where the peak yield is formed.

## 3. Constitutive Modelling

This section describes the constitutive polymer model that will be used in the RVE-based micromechanical model of UD composites from the following section.

### 3.1. Unified Semi-Crystalline Polymer (USCP) Model

**Figure 9:**
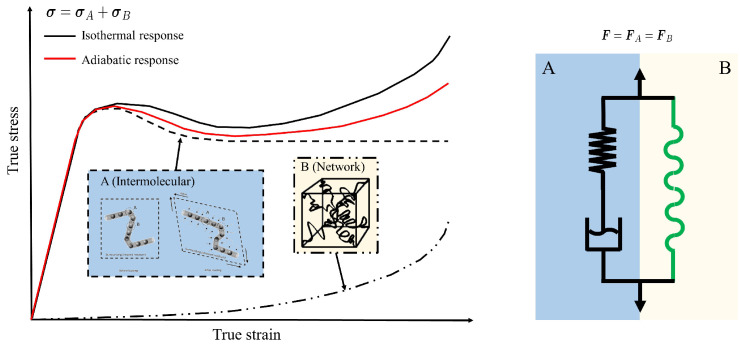
Schematic stress–strain representation of intermolecular and network resistances (**left**) and the corresponding rheological model (**right**).

The USCP model was developed according to the observation of the double yield phenomenon in SCPs [33,34,35], and it has been validated for a wide variety of SCPs such as nylon 101, LDPE and PA6 [29]. This model, formulated within the finite strain kinematic framework, is a generalization of the Boyce–Parks–Argon (BPA) model [32] by incorporating a single viscoplastic law that unifies both the amorphous and crystalline phases of the polymer. Although the USCP model was initially conceived for thermoplastics, it can also retrieve the response of the modified BPA (MBPA) model proposed by Poulain et al. [31] for thermosets as epoxy resins by just deactivating the crystalline phase. Figure 9 shows the rheological analogue and the corresponding behaviour.

The intermolecular and network resistances depicted in Figure 9 share the same deformation gradients in the assumed parallel arrangement, thus

(1) $$F_{A}=F_{B}=F.$$

The total Cauchy stress due to these contributions is given by

(2) $$\sigma =\sigma_{A}+\sigma_{B},$$

where $\sigma_{A}$ and $\sigma_{B}$ are the inter-molecular and network contributions, respectively.

The deformation gradient $F_{A}$ in branch A can be decomposed as follows:

(3) $$F_{A}=F_{A}^{e}F_{A}^{p},$$

where $F_{A}^{e}$ corresponds to the linear elastic response and $F_{A}^{p}$ is for the dashpot element.

Considering the velocity field v and the current coordinate system x, the Eulerian quantity velocity gradient $L_{A}=\partial v/\partial x$ in branch A is written as

(4) $$L_{A}={\dot{F}}_{A}{\left(F_{A}\right)}^{-1}=L_{A}^{e}+{\tilde{L}}_{A}^{p}=L_{A}^{e}+F_{A}^{e}L_{A}^{p}{\left(F_{A}^{e}\right)}^{-1},$$

where $L_{A}^{p}$ is the plastic velocity gradient expressed in the relaxed configuration, ${\tilde{L}}_{A}^{p}$ is the plastic velocity gradient in the loaded configuration, described by the rate of the inelastic contribution and it can be split into symmetric and skew parts, namely the inelastic rate of deformation ${\tilde{D}}_{A}^{p}$ and the inelastic spin tensor ${\tilde{W}}_{A}^{p}$, by assuming a spin-free plastic velocity gradient ${\tilde{W}}_{A}^{p}=0$:

(5) $${\tilde{L}}_{A}^{p}=F_{A}^{e}{\dot{F}}_{A}^{p}{\left(F_{A}^{p}\right)}^{-1}{\left(F_{A}^{e}\right)}^{-1}={\tilde{D}}_{A}^{p}$$

The Cauchy stress tensor in branch A $\sigma_{A}$ is obtained by eliminating the plastic deformation gradient $F_{A}^{p}$ updated from Equation (5).

The rate of inelastic deformation is written as

(6) $${\tilde{D}}_{A}^{p}=\dot{\bar{\varepsilon }}\;N,$$

where N is the direction tensor and $\dot{\bar{\varepsilon }}$ is the effective plastic strain rate. The latter one is derived by considering the thermally-activated double kink theory with free energy barrier, which is given by

(7) $$\dot{\bar{\varepsilon }}={\dot{\bar{\varepsilon }}}_{0}\exp\left[-\frac{A\left(s-\alpha \sigma_{h}\right)}{\theta }\left(1-{\left(\frac{\sigma_{\mathrm{eq}}}{s-\alpha \sigma_{h}}\right)}^{m}\right)\right],$$

where θ is the absolute temperature and $\sigma_{h}$ is the hydrostatic part of the Cauchy stress tensor. The material constants ${\dot{\bar{\varepsilon }}}_{0},\;m,\;A$ are the rate-dependent sensitivity parameters requiring parameter identification (PI) using the peak yield stresses at minimum two strain rates. The magnitude *s* is the so-called athermal effective stress and α is the pressure sensitivity parameter useful to capture tension-compression asymmetry. This work uses characterization only in compression, thus as a first approach α is set to zero. The driving stress is defined as the equivalent stress $\sigma_{\mathrm{eq}}$. The elastic modulus is assumed to follow a logarithmic form between the current and reference temperatures via the material constant β as follows:

(8) $$E\left(\theta \right)=\frac{E_{\mathrm{ref}}}{10^{\beta \left(\theta -\theta_{\mathrm{ref}}\right)}}.$$

The initial athermal effective stress $s_{0}$ is expressed as [31]

(9) $$s_{0}=\sqrt{3}\frac{8.5^{-1/m}}{\left(1-\nu \right)}\frac{E\left(\theta \right)}{2\left(1+\nu \right)}.$$

The evolution law for the athermal effective stress is formulated using a smooth, Heaviside-like function to characterize the pre-peak hardening, post-peak softening and second yield due to crystalline phase contribution, as follows:

(10) $$\dot{s}=H_{1}\left(\bar{\varepsilon }\right)\cdot \left(1-\frac{s}{s_{1}}\right)\cdot \dot{\bar{\varepsilon }}+H_{2}\left(\bar{\varepsilon }\right)\cdot \left(1-\frac{s}{s_{2}}\right)\cdot \dot{\bar{\varepsilon }}+H_{3}\left(\bar{\varepsilon }\right)\cdot \left(1-\frac{s}{s_{3}}\right)\cdot \dot{\bar{\varepsilon }},$$

where athermal strength $s_{i}$ (i = 1, 2, 3) corresponds to the preferred state at different stages [29]. It involves three hardening (softening) parameters, $h_{1}$, $h_{2}$ and $h_{3}$, the smoothing factor *f*, plastic strain at the peak yield, and a typical peak yield and two saturated states with athermal strength equal to $s_{1}$, $s_{2}$ and $s_{3}$, which are given as

(11) $$H_{1}\left(\bar{\varepsilon }\right)=-h_{1}\left\{\tanh\left(\frac{\bar{\varepsilon }-{\bar{\varepsilon }}_{p}}{f{\bar{\varepsilon }}_{p}}\right)-1\right\},$$

(12) $$H_{2}\left(\bar{\varepsilon }\right)=h_{2}\left\{-\tanh\left(\frac{\bar{\varepsilon }-{\bar{\varepsilon }}_{p}}{f{\bar{\varepsilon }}_{p}}\right)\tanh\left(\frac{\bar{\varepsilon }-{\bar{\varepsilon }}_{c}}{f{\bar{\varepsilon }}_{c}}\right)+1\right\},$$

(13) $$H_{3}\left(\bar{\varepsilon }\right)=h_{3}\left\{\tanh\left(\frac{\bar{\varepsilon }-{\bar{\varepsilon }}_{c}}{f{\bar{\varepsilon }}_{c}}\right)+1\right\},$$

where ${\bar{\varepsilon }}_{p}$ is the plastic strain at the peak yielding point, and ${\bar{\varepsilon }}_{c}$ is the characteristic plastic strain when the crystalline nano-block initiates to yield. In this work dealing with glassy amorphous polymer PR520, the crystalline phase related parameters are neglected (the third term in Equation (10)), which generalizes to the MBPA model [31].

The stress contribution of network resistance $\sigma_{B}$ depends on the rubbery modulus $C^{R}$ and the number of rigid links *N*. For more detailed constitutive modelling, readers are referred to Ref. [29].

### 3.2. Thermomechanical Coupling

Both thermoplastic PEEK and thermoset PR520 exhibit clear self-heating and thermal softening effects in the experimental results. Thermomechanical coupling is therefore required to capture these effects in the simulations. The heat balance equation was developed while taking into account the deformation associated plastic dissipation. The dissipation is assumed to be completely converted to heat. The heat balance equation is provided by assuming constant thermal specific heat $c_{p}$ and thermal conductivity *k*, which are given by

(14) $$\rho {\;c}_{p}\frac{\partial \theta }{\partial t}=\sigma_{A}:{F^{e}}_{A}{{L^{p}}_{A}\left({F^{e}}_{A}\right)}^{-1}+\nabla \cdot \left(k\frac{\partial \theta }{\partial x}x\right),$$

where ρ is the polymer density. The 2nd-order tensors ${F^{e}}_{A}$ and ${F^{p}}_{A}$ are the elastic and plastic deformation gradients on branch A, respectively. The plastic velocity gradient expressed in the relaxed configuration is denoted by ${L^{p}}_{A}$. More details on further considerations adopted for this coupling can be consulted in Ref. [29].

### 3.3. FEM Model of the High Speed Bench

Numerical simulations were conducted with and without self-heating effects to simulate both polymer responses obtained from the tests. To do this, a 3D FEM model has been developed to mimic the real setup described in the experimental section.

**Figure 10:**
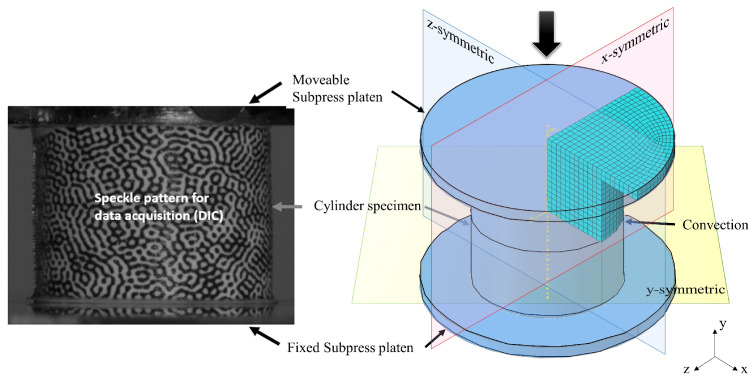
Cylinder specimen with friction-reducing sheets during high-speed compression (**left**) and one-eighth FE model with subpress platen (**right**).

As shown in Figure 10, symmetries permit us to work with a one-eighth FE model. The model incorporates the details of the metal subpress platen to attach the cylinder specimen. The specimen–platen contact is assumed to be frictionless. The thermal conductance of the metal platen is assigned to the contact surface with a value of 52 J/s m K. The specific heat and density of the platen were 420 J/kg K and 7800 kg/m${}^{3}$, respectively. Convection was applied to the outer curved surface with a value of 85 J/s m${}^{2}$ K [27].

To conduct a thermomechanical analysis, this FEM model uses temperature-displacement coupled elements. A mesh sensitivity study was previously conducted to ensure the mesh convergence based on the studies of Ref. [36], where the spatial and temporal discretization was studied for one-eight cylinder in compression. An approximated element length of 0.5 mm was proved to obtain the convergence. As boundary condition (BC), the platen is connected to a reference point via a rigid constraint. Thus, this point is enforced to move with the same speed as the real bench along the y-axis. The entire model has a predefined temperature field, and the temperature is updated by introducing the user-defined subroutine to solve the heat balance equation. Using volume-averaged homogenised values, the true stress–strain curve was obtained from the cylinder specimen.

### 3.4. Comparison FEM Versus Experiment

**Figure 11:**
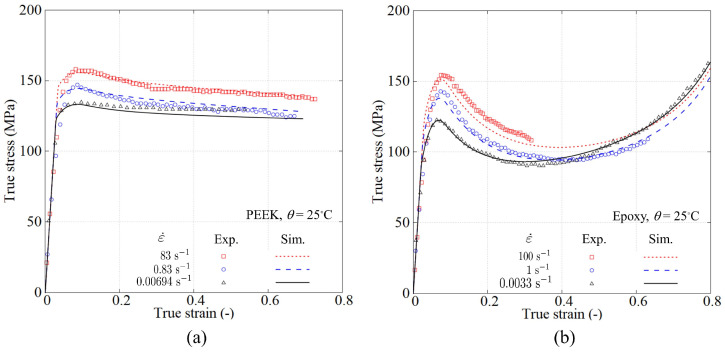
Comparison of the stress–strain curves between the model and the test at different strain rates: (**a**) PEEK KT880−NT and (**b**) PR520.

Figure 11 shows the comparison between experimental results (open symbols) and the FEM simulation using the presented constitutive polymer model under full thermomechanical coupling. To identify the material constants, the procedure described in [29] was followed. For the case of PEEK, this identification was performed at the lowest strain rate 0.00694 s${}^{-1}$, the result of which conforms perfectly with the experimental one. Self-heating and thermal softening effects are clearly observed from the experimental true stress–strain curves and the simulation can properly capture. It can be noticed that the saturated stress at 0.83 s${}^{-1}$ is even lower than the one at 0.00694 s${}^{-1}$, which leads to the crossing of two stress–strain curves. This feature is naturally captured by assuming the conversion of total plastic work to heat.

For the case of PR520, the identification procedure required the incorporation of the rubbery effect by introducing the network resistance described previously. The material constants $C^{R}$ and *N* were obtained using the experimental true stress–strain curve at 0.0033 s${}^{-1}$. The rubbery effect and the thermal softening are calibrated using two stress–strain curves at lower strain rates. Despite the stiffening caused by the network resistance, the model is able to give a good prediction at the highest strain rate (100 s${}^{-1}$). The USCP model was originally conceived for thermoplastics; however, these results demonstrate its applicability to advanced epoxy-based resins such as PR520 over a wide strain rate range.

### 3.5. Predicted Temperature Profile in Quasi Static-to-Dynamic Regime

**Figure 12:**
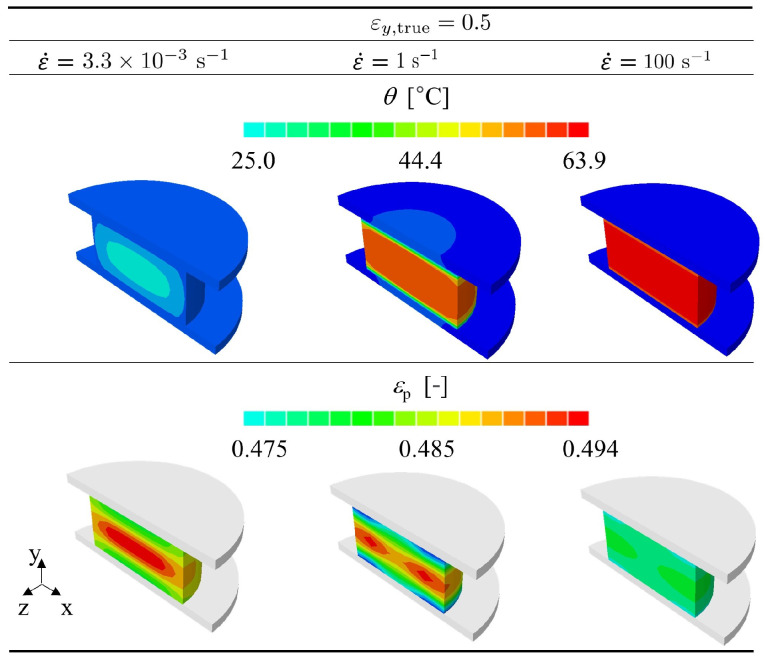
Predicted temperature distribution within the PR520 cylinder specimen at different strain rates.

This section shows how the proposed model can assess the distribution of temperature inside the specimen under different loading rates. Since both polymers gave the same qualitative response in terms of heat profiles, only the case of PR520 is shown to illustrate the model prediction. Figure 12 shows how the temperature (top) and the equivalent plastic strain (bottom) are spatially distributed in the specimen when the true strain is 0.5.

It can be appreciated that the contour plots of the equivalent plastic strain are consistent with the temperature field. At lower strain rates, the specimen develops a hot core that is slowly dissipated to the exterior due to the slow test duration. On the other hand, the equivalent plastic strain varies less at higher strain rates because the corresponding temperature field in a nearly adiabatic condition results in more homogeneous thermal softening at each material point.

## 4. Application to UD Composite

This section introduces the RVE micromechanical model to investigate the response of UD composites via FEM. The corresponding material properties are assigned to each constituent: fibre, matrix and interface (refer to Appendix A). The validated USCP model is used here to represent the two characterized polymers, namely, PR520 and PEEK KT880−NT. This RVE-based UD composite model serves as a tool to assess the differences obtained when a thermoplastic resin is used in comparison with a thermoset resin. Accordingly, the overall thermomechanical response of the UD model is examined under high-speed transverse compression at different strain rates. In addition, this study incorporates the possibility of interface debonding in both material systems, in order to evaluate the loss of mechanical performance.

Various types of boundary conditions (BC) are examined in comparison to the reference case established by the Periodic Boundary Condition (PBC) [22]. Since the constitutive model is thermomechanically coupled, self-heating and thermal softening effects occurring in the matrix can be assessed in the UD model.

### 4.1. RVE Micromechanical Model

**Figure 13:**
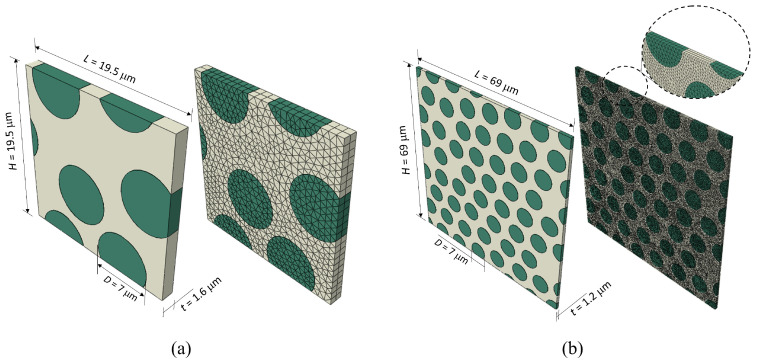
Geometry and mesh of the microscale RVE models used in this work: (**a**) VF40% with 4 fibres and (**b**) VF40% with 50 fibres.

Figure 13 shows the geometry of the 3D microscale RVE models representing a UD composite consisting of an initially squared matrix domain with embedded carbon fibres. The fibres were randomly distributed in the matrix using a fibre packing generator adapted from the research develop in Ref. [37]. This generator assumes periodic boundary conditions during the fibre positioning and it equally adjusts the lateral dimensions of the RVE according to the available number of fibres and the desired fibre volume fraction (VF).

In this work, two types of RVE are used. The first one, represented in Figure 13a, is an RVE with a fibre volume fraction of 40% containing 4 fibres, which is deemed practical to analyse the BC effects. The second one, represented in Figure 13b, is a larger RVE also with 40%, but containing 50 fibres. This bigger model is used to perform the thermomechanical analysis under fast transverse compression. Both RVE models have a similar thickness containing more than two elements along the fibre direction (see Figure 13). Fibres and matrix are meshed using C3D6T wedge elements [38] after conducting a mesh convergence analysis to guarantee consistent results. The allowable maximum element size was approved as 0.8 µm.

A fibre diameter of 7 µm is chosen according to the typical size of carbon fibres. The fibres are assumed as a transversely isotropic linear elastic material, whose material properties have been taken from Ref. [25]. This work analyses transverse loading, which makes the fibre failure unlikely, therefore fibre damage was not incorporated in the model. Constant thermal properties along and perpendicular to the fibre are assumed [39].

As mentioned, a fully thermomechanical coupling analysis has been conducted. In order to characterize the mechanical and thermal responses, two user-defined material behaviours were integrated. A temperature field of 23 °C was predefined for the entire RVE model. The only source of heat is generated from the polymer matrix, where the plastic deformation takes place. The plastic dissipation is estimated at each time increment and provided to the thermal model. The heat balance equation is solved in order to update the new temperature field. The thermal softening occurs in the subsequent time increment, and the mechanical properties of the material are changed accordingly to the predetermined temperature-dependent relations. The thermal conductivity was assigned to the fibre–matrix interface for allowing the exchange of heat between the matrix and the fibres. The thermal gap conductance coefficient of the interface is set as the same as matrix. With this assumption, the ability to conduct the heat across the interface can be modelled when the separation is within a gap tolerance.

### 4.2. Fibre–Matrix Interface

**Figure 14:**
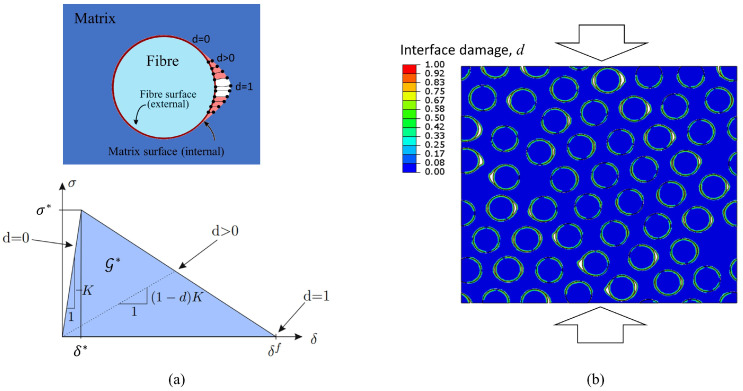
(**a**) Traction-separation response at the interacting surfaces of the fibre–matrix interface. (**b**) Multiple fibre debonding taking place under compression in a RVE with 50 fibres (scale factor 2).

The strength of the fibre–matrix interface can play an important role in the overall composite strength, therefore this feature has been explicitly incorporated in the RVE model. The interface between the fibres and the matrix has been modelled using a surface-based cohesive behaviour that follows the bilinear traction-separation law schematically represented in Figure 14a. A stress-based failure criterion is used to determine the onset of the fibre debonding. This approach prescribes the interfacial normal and shear tractions that resist the separation and the relative sliding at the interface.

The traction, integrated to complete separation, yields the fracture energy release rate, $G^{*}$. The cohesive length of the interface $\delta^{f}$ is defined as $\delta^{f}=2\;G^{*}/\sigma^{*}$, where $\sigma^{*}$ is the interface bonding strength. The variable *d* is a scalar between 0 and 1 that quantifies the deterioration of the cohesive interaction between the fibre and the matrix. Figure 14b shows an example of the damage variable when multiple fibre debonding takes place under compression in a RVE with 40% of fibre volume fraction.

It is worth mentioning that the positioning of the fibres with respect to the applied load can generate a mixed-mode stress state in the cohesive interface. Therefore, the debonding mode mixity has to involve different energies associated to the debonding capability in normal and parallel directions to the interface. To do this, this work assumes that the rate of debonding progression is controlled by the critical energy release rate according to the Benzeggagh–Kenane (BK) law [40].

This description makes it possible to describe full contact, under compression, tension and shear, between fibres and the surrounding matrix. More details of this contact formulation can be seen in Ref. [41]. The properties of the fibre–matrix interface have been obtained from Ref. [25].

### 4.3. Boundary Conditions

Periodic Boundary Conditions are widely used in RVE-based micromechanical modelling to study a broad variety of composite materials [42,43]. This allows researchers to study the material as if it was a large system (i.e., macroscopically), but with direct access to its microstructure. However, this approach is debatable when a damage process is involved in any of its microstructural constituents. Assuming a periodic RVE also means that the failure will be periodically taking place along the whole extension of the bulk material. This situation might overestimate the influence of damage in the overall response. Additionally, from a computational point of view, PBC also involves a much larger and constrained system in the finite element calculation, leading to more numerically intensive calculations compared to using uniform or homogeneous boundary conditions [22]. There are more advanced PBCs in which the temperature is also involved, similar to the ones described in Refs. [39,44]. For the sake of simplicity, this work does not consider periodicity in the temperature field.

Based on the results of Ref. [45], the present investigation looks for an alternative to PBC, which can be computationally more feasible and circumventing the periodic enforcement of any possible source of damage. As also stated in Ref. [46], by increasing the size of the microstructural domain, the convergence of the overall response will take place by sufficiently large domains.

**Figure 15:**
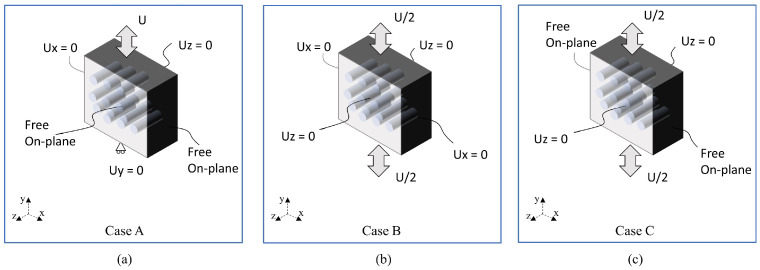
Non-periodic boundary conditions explored in this research: (**a**) Case A applies tensile load as in a one-eight model, (**b**) Case B applies tensile load in a transversally constrained model, and (**c**) Case C applies tensile load as in a plane strain model.

For this aim, the RVE with four fibres shown in Figure 13a is used to test the effect of the boundary constraining. Four cases have been studied, one periodic and three non-periodic (Figure 15) and their assumptions are explained as follows:

- Case PBCs: This is the first case corresponding to the classic PBC and implemented as described in Ref. [22]. This case is considered here as the reference case.
- Case A: The RVE is uniformly and vertically loaded from the top surface, whilst the vertical displacement of the bottom surface is constrained. With respect to the lateral surfaces, in the surface pair perpendicular to the x-axis, one of the surface has blocked its movement along the x-axis. However, the other surfaces can move freely along the x-axis, but assuming that both the nodes of the fibre and the matrix are always contained in the same plane to prevent the possibility of fibre push-out when the interface is totally debonded. This condition is hereafter referred to as “Free On-plane” condition. It can be seen that Case A is very similar to considering a one-eighth model, in which the planes OX, OY and OZ are considered as symmetry planes.
- Case B: Here, the load is imposed symmetrically from the top and the bottom surfaces, but restricting the lateral displacement of the RVE. This case is highly constrained and it is used to establish an upper limit in the stress–strain response.
- Case C: this case also applies the load symmetrically, but the lateral displacement of the surfaces perpendicular to the x-axis are considered as “Free On-plane” conditions. However, the displacements of the surfaces perpendicular to the fibre direction, namely the z-axis, are set to zero. This condition can be seen as an equivalent case to the plane strain condition. Since the fibres are very stiff, in case of transverse compression, it is reasonable to assume that the in-plane displacements will be negligible compared to the transverse ones.

With respect to output provided from this RVE model, apart from the direct access to the microscopic details, the overall stress–strain response can be obtained via first order homogenization, which is deemed sufficient when conducting axial load [46]. The engineering measures were calculated directly by the force and displacement from the reference point.

Three typical polymers for UD composites were used: a semi-crystalline polymer PA6 presenting the double yield phenomenon, a PR520 exhibiting a rubbery effect and a high performance thermoplastic PEEK. All stress–strain responses were characterized using the USCP model according to their different features, and the identified parameters are provided in Table A1.

**Figure 16:**
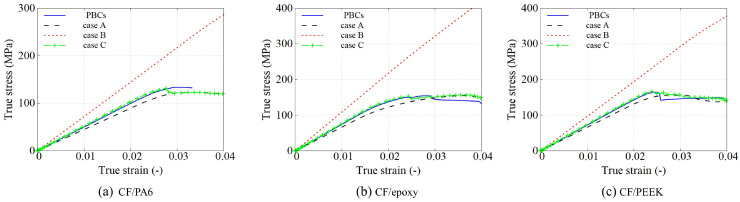
Comparison of true stress–strain curves of 4 different types of BCs using three popular UD composites at the strain rate of 100 s${}^{-1}$.

Figure 16 shows the comparison between the described boundary cases. It can be noticed that Case A establishes a lower limit in the stress–strain response as it is assuming fully free-moveable lateral surfaces leading to the most unconstrained case. On the opposite side, Case B generates the stiffest results according to the level of mechanical restriction, which was already expected. With respect to the Case C, it is interesting to see how it generates a very similar response as assuming a three-dimensional PBC.

**Figure 17:**
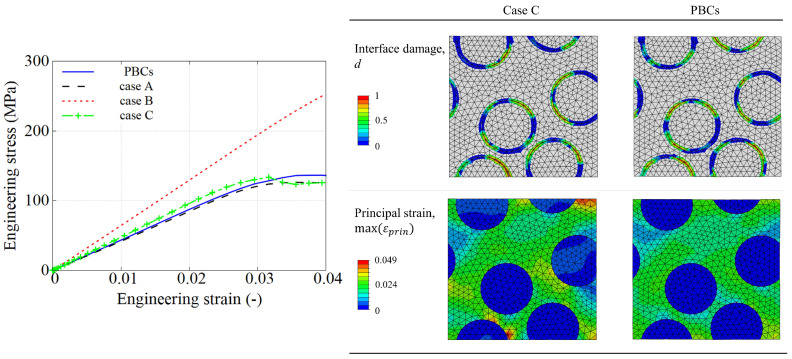
Comparison of four types of BCs at a strain rate of 100 s ${}^{-1}$. (**Left**) Stress–strain response considering engineering values. (**Right**) Interface damage and maximum principal strain field at a strain value of 0.035.

In order to assess this comparison, engineering stress–strain definitions have been also plotted for CF/PA6. Figure 17 shows that the stress–strain conformity between Case C and Case PBCs is slightly larger. The interface damage profiles are very similar in both cases at a chosen strain level of 0.035, as well as the local strain fields. It is worth mentioning that this similar response between both cases already occurs in a relatively small RVE. The RVE is more constrained on the border in Case C, showing a higher strain level compared to the Case PBCs counterpart. As a consequence, using a larger RVE will certainly decrease the differences, where the dissimilar local values in stress and strain are less noticeable in the averaged overall response.

In terms of the computational efficiency, Table 1 shows that Case PBCs overwhelms other non-PBCs cases, lasting 19 h for a RVE model of only four fibres using a work station equipped with Intel(R) Xeon(R) E5-2667 processor and 128 GB of RAM, whereas the calculation of non-PBCs cases requires less than one hour. This result suggests that Case C can be used as a doable and representative boundary condition to study the response of the large RVE of UD composite under fast compression load. Therefore, the following sections will assume Case C to study the thermomechanical response of a RVE with 50 fibres shown in Figure 13b.

## 5. Model Prediction of UD Composites under High Compression Rate

This section compares the response of both UD composite systems, namely, CF/epoxy using the PR520 resin and the CF/PEEK using the KT880−NT resin. The effect of strain rate, fibre–matrix debonding and the influence of the local self-heating in the composite matrix due to short testing time will be discussed. The matrix damage is not considered in this paper.

### 5.1. Stress-Strain Response: General Assessment

**Figure 18:**
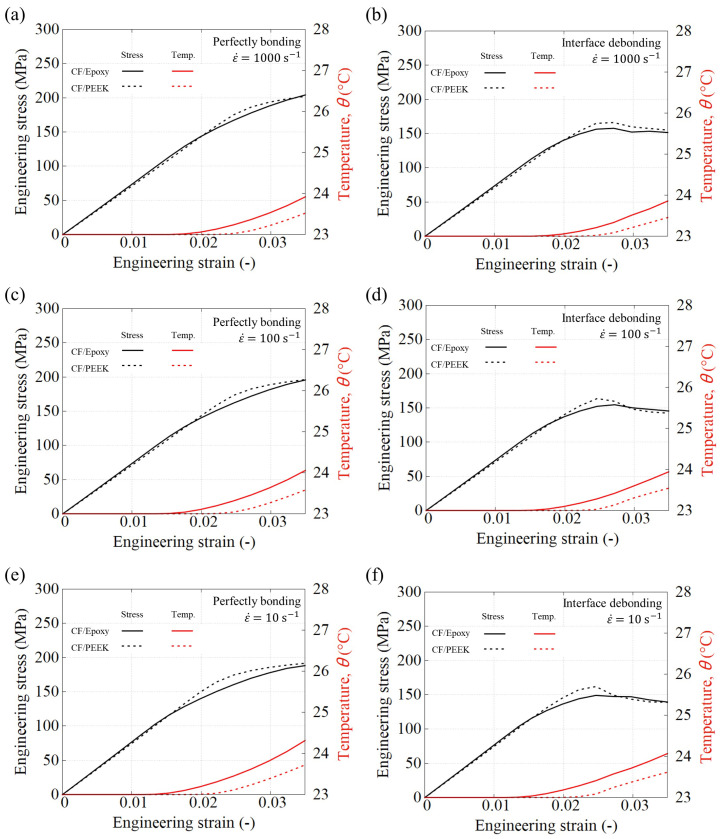
Comparison of the CF/PR520 and CF/PEEK systems under compression at three high strain rates: 10 s${}^{-1}$, 10${}^{2}$ s${}^{-1}$ and 10${}^{3}$ s${}^{-1}$. The effect of the fibre–matrix interface is also compared: (**a**,**c**,**e**) without debonding, (**b**,**d**,**f**) with debonding.

Figure 18 shows a general layout obtained from the RVE-based UD model in terms of stress–strain and temperature–strain curves. The simulations show that both composite systems exhibit very similar behaviours. Additionally, the influence of assuming perfect or imperfect fibre–matrix debonding is similar in both systems. In that sense, it must be recalled that the commercial PEEK KT880−NT has been formulated to achieve a similar response as the toughened thermoset PR520, both in terms of stiffness and overall strength. It can also be noted that, although the PEEK matrix is a semi-crystalline thermoplastic, this characteristic does not seem to have any visible influence in the studied range of strain, strain rate and temperature-related response.

Figure 18 also depicts the evolution of the average temperature in the composite as a function of the strain. The temperature increase takes clearly place once after the polymer matrix has exceeded its plastic yield point, which is approximately identifiable at the moment where there is a deviation of the linearity in the stress–strain curve. In terms of temperature increase, the CF/PR520 system seems to generate systematically larger values, as well as showing a faster rate after the yield point.

In both composite systems, the influence of the fibre–matrix debonding is remarkable. If debonding takes place, both types of composites exhibit a clear stress peak followed by a stress drop. Remind that no matrix damage is considered in this work, therefore, such a drop might be different in case of localized matrix failure. In that sense, if the matrix fails (either in a plastic or brittle fashion), the fibre–fibre contact might still carry the compressive load wherein the “crushed” matrix is located in between.

### 5.2. Strain Rate Effect

From the previous Figure 18, the effect of the strain-rate is not totally obvious. In that sense, a more pragmatic way to assess such an effect has been considered in this work. To do this, the area enclosed below the stress–strain curve has been calculated for each case. The strain range to compute this area has been the same for all the cases and it lies between 0 and 0.035. According to the common values of strain at failure in UD composites under transverse compressive load (4–5%, see for example Ref. [5]), the range 0–3.5% is deemed sufficiently representative to give a good idea of how the strain rate could affect the performance before drastic failure happens.

**Figure 19:**
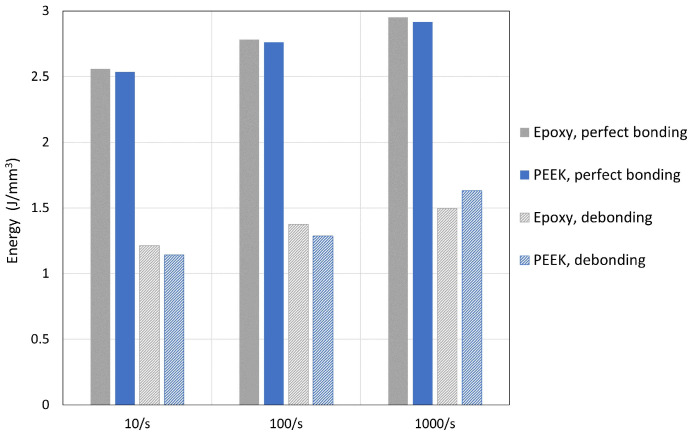
Energy per unit volume in both material systems under different strain rates with and without fibre–matrix debonding.

Figure 19 is organized in three sets of bars charts corresponding to the strain-rate value. In each set, the two composite systems are compared under two situations, namely, without and with fibre–matrix debonding. “Perfect bonding” refers to a permanent fibre–matrix bonding, whose bars are depicted with a solid colour. In case of debonding, dashed colours are used instead. The plotted quantity can be somehow interpreted as a quantitative measure of the energy absorption capability that such a composite would have under compressive load. This quantity represents an energy per unit volume (J/mm${}^{3}$) and it might also be interpreted as a measure of the toughness under a compression-dominated impact. It is clear that the debonding of fibre drastically reduces the material resistance by almost a factor of 2.

Again, both composite systems behave very similarly. It is worth mentioning that a UD composite based on the specially formulated epoxy PR520 behaves similar to a high performance thermoplastic-based UD composite. In both systems, the energy per unit volume is augmented as the strain rate increases. The only difference, perhaps, can be observed in the change of trend in the case of debonding. Under 10 s${}^{-1}$ and 100 s${}^{-1}$, the system CF/PR520 performs slightly better than CF/PEEK. However, this trend inverts at the highest strain rate, making the CF/PEEK slightly more resistant. In any case, these differences are only indications and they must be taken with caution until a more detailed model including matrix damage is studied.

### 5.3. Fibre Debonding Effect

**Figure 20:**
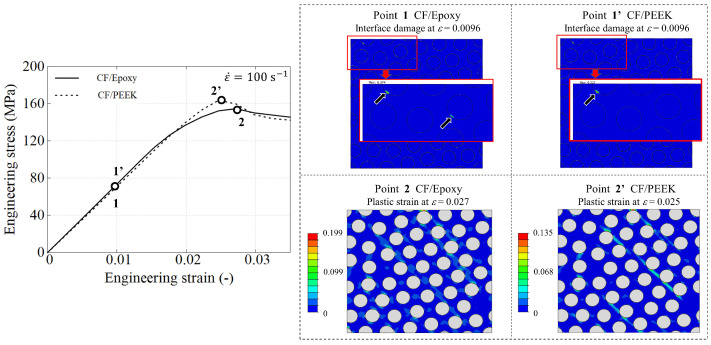
Stress−strain behaviour of CF/PR520 and CF/PEEK systems at two representative points: onset of debonding and at peak stress.

Figure 20 presents a correlation between the overall stress–strain response and the UD microstructure for both composite systems. A strain rate of 100 s${}^{-1}$ has been used as a reference, which fits the characteristic rate well in practical situations such as vehicle crashes, where the materials can deform at rates up to 200 s${}^{-1}$ [47]. In this figure, two snapshots of the model are extracted during the loading process. The first point, labelled by 1 for the CF/PR520 system (and $1^{\prime }$ for CF/PEEK) determines the instant of the initiation of fibre debonding. The second point, labelled by 2 (or $2^{\prime }$), indicates the instant at which both composite systems have reached the peak stress.

The onset of debonding is very similar in both composite materials, even in the physical location. The RVE model predicts that the CF/PR520 system generated a slightly lower peak stress in which the plastic strain localization reaches almost 20%. The CF/PEEK system gives a slightly higher peak stress at a slightly lower strain, but developing lower plastic strain localization.

From these results, two important points can be highlighted. Firstly, it is clear that the interface damage plays a very important role in the nonlinear overall response in the strain range between 0.01 and 0.025. In other words, this nonlinearity is not exclusively due to the viscoplasticity of the polymer matrix. Second, the results show high local plastic deformations already at a relative small global strain in which the rate-sensitivity of UD composite is governed by the rate-dependence of the polymer matrix.

### 5.4. Self-Heating Influence: Local vs. Global Effect

**Figure 21:**
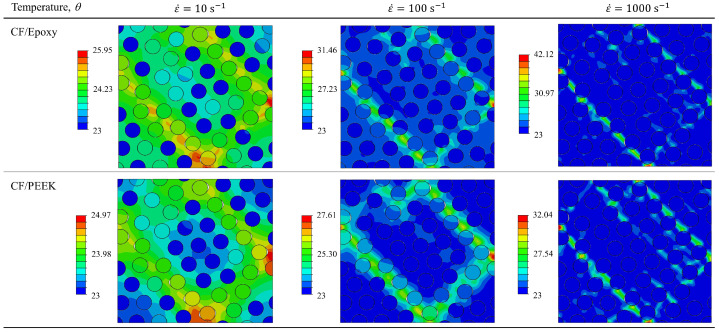
Temperature profiles of CF/PR520 and CF/PEEK systems at three high strain rates and a strain level of 0.035.

The previous Figure 18 has shown that the average temperature at low strain rate is higher than at the highest strain rate. In order to investigate how the temperature distributes in the UD composite, Figure 21 shows the temperature profile in both composite systems at three strain rates. These snapshots correspond to a value of applied strain equal to 0.035. At a lower strain rate, a wider testing time period allows the heat diffusion more easily, leading to a more homogeneous thermal softening. In other words, the dissipated energy from the plastic deformation is spatially distributed everywhere, permitting it to heat up the composite material more globally. On the other hand, the temperature field becomes more localized when the strain rate increases, where the plastically-driven shear bands also become sharper. An increasing strain rate favours the localization of plastic deformation in narrower bands, promoting the punctual matrix thermal softening. However, the average temperature at lower strain rates remains still higher.

## 6. Conclusions

This work presents a complete work-flow for the thermomechanical modelling of UD composites under high-speed transverse compression. The main contribution covers two main aspects: from the robust high-speed characterization and advanced physically-based modelling of different thermosets and thermoplastics in a pure state to their applications in UD composites.

- In-house high-speed compression tests were performed for a toughened epoxy PR520 and a semi-crystalline thermoplastic PEEK. Three-dimensional stereo DIC and infrared camera systems were integrated to investigate the rate-sensitivity and self-heating effect.
- An advanced glassy polymer model (USCP) was used to characterize the rate-dependence of different thermosets and thermoplastics. The rubbery effect, double yield of SCPs, and thermal softening were accurately predicted for various polymers.
- The rate-dependence of UD composites is naturally captured by applying the USCP model for polymer matrices. The numerical results of the RVE analyses show that the nonlinear response in the small strain range (ε<0.04) is attributed to the synergy of the viscoplastic behaviour of the polymer matrix and the interface debonding.
- An alternative boundary condition was used, which provides a similar result compared to the reference case using PBCs. This BC is proved to be very computationally efficient for the case of an adequately large RVE.
- Both CF/PR520 and CF/PEEK systems experience an excessive strain localization. A large local plastic strain up to 0.19 can be found in the matrix at a macroscopical strain level of 0.035.
- Insights of the self-heating and thermal softening induced by the polymer matrix on the global response were provided. The average temperature at a lower strain rate remains higher and more homogeneous thanks to the long testing period allowing the heat diffusion throughout the RVE.

To achieve a high fidelity modelling of thermosets and thermoplastics, accurate measurements of the temperature-dependent thermal properties are also recommended. The experimental validation of the rate-dependence of UD composites will be conducted in a future publication.

## Figures and Tables

**Table 1:** Comparison of CPU time (4 CPUs) in second using different cases for RVE model with 4 fibres.

Case	A	B	C	PBCs
CF/PA6 ${}^{1}$	754	729	772	21508 ^†^
CF/PR520 ${}^{1}$	744	714	748	35137
CF/PEEK ${}^{2}$	1170	1118	1181	71579

^1^ Intel(R) Xeon(R) Gold 6146 processor and 256 GB of RAM. ^2^ Intel(R) Xeon(R) E5-2667 processor and 128 GB of RAM. ^†^ Termination due to the excessive distortion of element.

## Data Availability

Provided upon request.

## Appendix A. Thermomechanical Properties and Identified Model Parameters

The appendix summarizes the input parameters for the thermomechanical analyses of UD composites. Tables are provided separately for each constituent, including the elasto-viscoplastic response of matrices, elastic response of carbon fibre and the traction-separation law of fibre–matrix interface. Readers are referred to the calibrated parameters for USCP model in Table A1 with the corresponding thermal properties for the investigated polymers used in this study.

**Table A1:** Material parameters for the investigated PA6 [29], PEEK [30] and PR520 [30].

Material Parameter	Unit	PA6	PEEK	PR520	Description
Mechanical constants					
$E_{\mathrm{ref}}$	GPa	2.62	4.33	4.28	Modulus at $\theta_{\mathrm{ref}}$
$\theta_{\mathrm{ref}}$	K	296	298	298	Reference temperature
ν	-	0.39	0.37	0.40	Poisson’s ratio
β	1/K	0.0036	0.0007	0.0012	Temperature dependence
α	-	0	0	0	Pressure sensitivity
${\dot{\bar{\varepsilon }}}_{0}$	1/s	3.55 × 10^11^	4.27 × 10^4^	1.76 × 10^9^	Rate sensitivity
*m*	-	0.83	0.66	0.66	Rate sensitivity
*A*	K/MPa	104	177.4	133.8	Rate sensitivity
$s_{0}$	MPa	184	161.6	172.3	Initial equivalent strength
$s_{1}$	MPa	196	174.8	242.5	Athermal peak strength
$s_{2}$	MPa	193	169.3	179.5	First saturation strength
$s_{3}$	MPa	234	-	-	Second saturation strength
$h_{1}$	MPa	32351	3747	6798	Pre-peak hardening
$h_{2}$	MPa	14827	959	635	Post-peak softening
$h_{3}$	MPa	681	-	-	Second yield hardening
${\bar{\varepsilon }}_{p}$	-	0.009	0.0566	0.0407	Peak plastic strain
${\bar{\varepsilon }}_{c}$	-	0.0295	-	-	Activation plastic strain
*f*	-	0.3	0.3	0.3	Smooth factor
$C^{R}$	MPa	-	-	13.5	Rubbery modulus
*N*	-	-	-	1.7	number of rigid links
Physical constants					
ρ	kg/m${}^{3}$	1200	1300	1250	Density
*k*	W/m K	0.25	0.25	0.192	Thermal conductivity
$c_{p}$	J/kg K	1700	1330	1100	Specific heat

**Table A2:** Transversely isotropic elastic properties [25] and thermal [39] properties of the carbon fibre.

Density	Mechanical					Thermal		
ρ (kg/m${}^{3}$)	$E_{11}$ (GPa)	$E_{22}$ (GPa)	$\nu_{12}$	$G_{12}$ (GPa)	$G_{23}$ (GPa)	$k_{∥}$ (W/m K)	$k_{⊥}$ (W/m K)	$c_{p}$ (J/kg K)
1800	276	15	0.2	15	7	10.2	1.256	750

**Table A3:** Properties of the fibre–matrix interface used in the thermomechanical analyses [25].

Strength			Critical Energy Release Rates			Mixed-Mode (BK Law)
$\tau_{1}^{0}$ (MPa)	$\tau_{2}^{0}$ (MPa)	$\tau_{3}^{0}$ (MPa)	$G_{\mathrm{IC}}$ (N/mm)	$G_{\mathrm{IIC}}$ (N/mm)	$G_{\mathrm{IIIC}}$ (N/mm)	η
75	75	50	0.002	0.006	0.006	1.45
